# Alleviation of cisplatin‐induced hepatotoxicity by gliclazide: Involvement of oxidative stress and caspase‐3 activity

**DOI:** 10.1002/prp2.788

**Published:** 2021-05-18

**Authors:** Fatemeh Taghizadeh, Seyed Jalal Hosseinimehr, Mehryar Zargari, Abbasali Karimpour Malekshah, Mansoureh Mirzaei, Fereshteh Talebpour Amiri

**Affiliations:** ^1^ Department of Anatomy Faculty of Medicine Molecular and Cell Biology Research Center Mazandaran University of Medical Sciences Sari Iran; ^2^ Student Research Committee Faculty of Medicine Mazandaran University of Medical Sciences Sari Iran; ^3^ Department of Radiopharmacy Faculty of Pharmacy Mazandaran University of Medical Sciences Sari Iran; ^4^ Department of Biochemistry Faculty of Medicine Mazandaran University of Medical Sciences Sari Iran

**Keywords:** caspase‐3, cisplatin, gliclazide, hepatotoxicity, oxidative stress

## Abstract

**Aims:**

Cisplatin (CP), as an effective alkylating agent, is widely used in cancer treatment, while hepatotoxicity is one of its side effects. Gliclazide (GLZ), as an oral hypoglycemic drug, has antioxidant and anti‐inflammatory properties. This study was designed to investigate the protective effect of GLZ against CP‐induced hepatotoxicity in mice.

**Methods:**

In this experimental study, 64 adult male mice randomly were allocated into eight groups (8 mice/group). Control, GLZ (5, 10, and 25 mg/kg, orally), CP (10 mg/kg, single dose, intraperitoneally), and CP+GLZ (in three doses). GLZ was administrated for 10 consecutive days. CP was injected on the 7th day of the study. At the end of the experiment, hepatotoxicity was evaluated by serum and tissue biochemical, histopathological, and immunohistochemical assessments.

**Results:**

The data were revealed that CP increased oxidative stress (increased MDA and reduced GSH), liver damage enzymes (ALT, AST, and ALP), and immunoreactivity of caspase‐3 in liver tissue of CP‐injected mice. Also, CP induced histopathological changes such as eosinophilic of hepatocytes, dilatation of sinusoids, congestion, and proliferation of Kupffer cells. GLZ administration significantly ameliorated serum functional enzyme and hepatic oxidative stress markers in CP‐injected mice. In addition, the histological and immunohistochemical alterations were ameliorated in GLZ‐treated mice. Of the three doses, 10 and 25 mg/kg were more effective.

**Conclusions:**

In conclusion, GLZ with its antioxidant, anti‐inflammatory, and anti‐apoptotic activities, can be suggested as a promising drug in the treatment of CP‐induced hepatotoxicity.

## Graphical abstract

An experimental study that evaluated the effect of gliclazide on cisplatin‐induced hepatotoxicity.

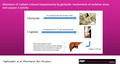

## 1 INTRODUCTION

Cisplatin (cis‐diamminedichloroplatinum; CP) is an effective alkylating and antineoplastic agent that is widely used in the treatment of solid tumors.^1^ The liver is a key organ in the metabolism and detoxification of endobiotics and xenobiotics.^2^ CP is accumulated in liver cells, leading to hepatotoxicity. The increased oxidative stress and inflammation are the main mechanisms involved in hepatotoxicity induced by CP.^3^ Furthermore, CP affects the tumor‐suppressor protein p53 through the generation of reactive oxygen species (ROS) and induces apoptosis via intrinsic caspases.^4^ Loss of liver histoarchitecture, positive caspase‐3 reactions, decrease in GSH, and an increase in MDA levels were reported in CP treatment.^2^ Since oxidative stress is the most important mechanism of the induction of hepatotoxicity, the use of antioxidants can play a major role in reducing CP‐induced toxicity.^5^

Gliclazide (GLZ) as an oral hypoglycemic drug and a second‐generation sulfonylurea, is used in diabetic patients.^6^ GLZ is able to improve the histopathological changes and DNA damage underlying both the process of aging and type 2 diabetes by reducing free radical generation and an increase in free radical scavenging.^6^ In addition to its antioxidant property, GLZ has potentially prevented tissue damage by acting anti‐apoptotic and anti‐inflammatory effects.^7^, ^8^ In diabetic experimental models, it has been demonstrated that GLZ preserves brain and sciatic injury ^9^ and DNA damage in peripheral blood lymphocytes.^10^ Only one study reported the synergistic effect of metformin and GLZ on liver injury in diabetic patients.^11^ And until now, no study reported the protective effect of GLZ on CP‐induced hepatotoxicity. Two case studies reported that GLZ at high doses causes acute hepatitis.^12^, ^13^ However, several studies have shown that low‐dose GLZ with having antioxidant properties mitigates DNA damage induced by Type 2 diabetes mellitus (T2DM) patients.^14^ With this background, in this study, we have investigated the effects of GLZ on oxidative stress, apoptosis, and histopathological changes induced by CP on liver tissue.

## 2 MATERIALS AND METHODS

### 2.1 Reagents

Cisplatin (1 mg/mL; Oncotec Pharma Production Gmbh‐ALLEMAGNE, code: 5622539, Germany) was got from a pharmacy. Primary antibodies of caspase‐3 and a secondary antibody conjugated with horseradish peroxidase were purchased from Abcam. Gliclazide was from a Pharmaceutical Co. (Tehran Daru).

### 2.2 Animals and experimental design

In this experimental study, 64 male BALB/c mice (25–30 g weighting) were obtained from the Animal Research Center of Mazandaran University of Medical Sciences, Sari, Iran. All experimental procedures were approved in accordance with the Institutional Animal Ethics Committee of the Mazandaran University Medical Sciences (ID: IR.MAZUMS.REC.1398.6106). The study was conducted in accordance with the Basic & Clinical Pharmacology & Toxicology policy for experimental and clinical studies.^15^ The animals were kept under standard laboratory conditions (23 ± 2°C, 55 ± 5% moisture, 12:12 h light/dark cycle) with free access to food and water.

**FIGURE 1:**
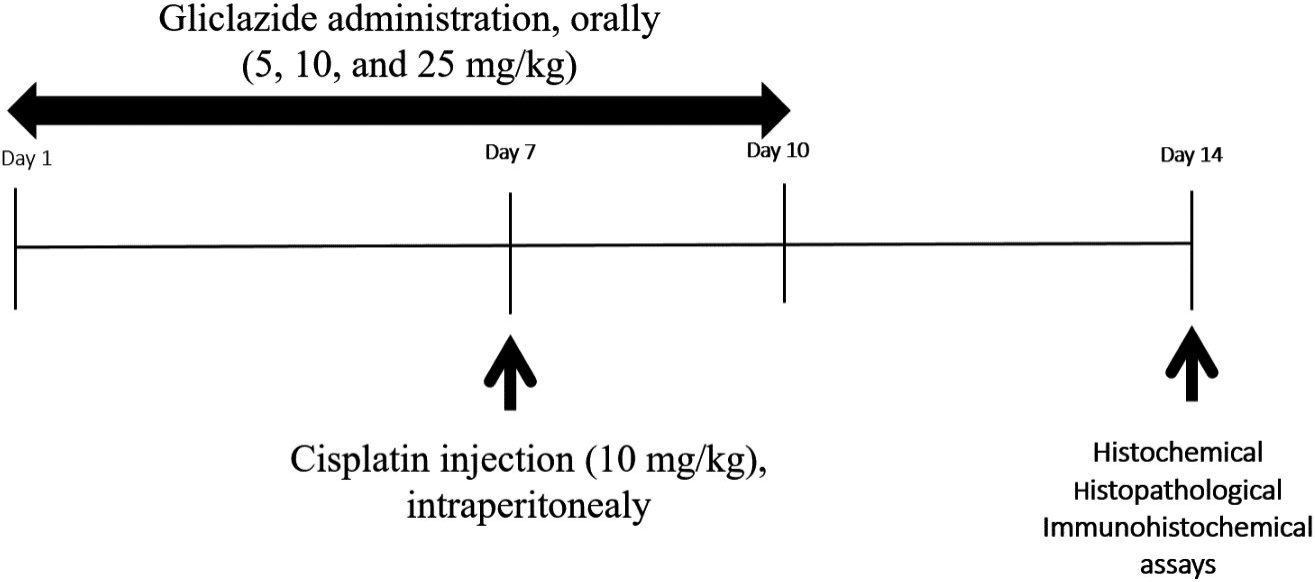
Study design diagram for protective effect of gliclazide (GLZ) on cisplatin (CP)−induced hepatotoxicity

In this study, animals were randomly divided into eight groups (8 mice/group). Groups were defined as follows: Group I; as control animals received distilled water (vehicle of GLZ). Group II, III, and IV; animals were administered with GLZ at doses of 5, 10, and 25 mg/kg/day for 10 consecutive days orally. Group V; hepatotoxicity was induced in mice by a single‐dose intraperitoneal injection of CP (10 mg/kg/day) on the 7^th^ day. Groups VI, VII, and VIII were administered GLZ at three doses of 5, 10, and 25 mg/kg via oral administration for 10 consecutive days, and CP injected on the seventh‐day study. The doses of CP chosen were based on our previous study ^16^ and GLZ based on the basis of the literature studies.^9^ A study design scheme is represented in Figure 1.

### 2.3 Sample collections

One week after receiving the last drug, the animals were euthanized with ketamine (50 mg/kg) and xylazine (5 mg/kg). Immediately blood samples were collected from the heart using a heparin syringe, then poured into the gel clot tube. After 15 min of centrifugation at 3000 × g for 15 min, the serum samples were separated and stored at −20°C for serum biochemical evaluation. Then, immediately the liver was carried out. One part of the liver was fixed in a 10% buffer formalin solution for histological and immunohistochemical examination and another piece of liver was washed in PBS, weighed, and then freshly frozen and stored at −70°C for biochemical analysis.

### 2.4 Liver function analysis

Aspartate aminotransferase (AST), alanine aminotransferase (ALT), and alkaline phosphatase (ALP) are liver function enzymes in the serum that were measured by Azmoon Co. kit according to the manufacturing instructions (1 400 018; AST, 1 400 019; ALT and 12,201; ALP). The liver enzyme level was recorded as U/L.

### 2.5 Oxidative stress assay

The liver samples were homogenized in cold phosphate buffer, centrifuged, and the supernatants were used for oxidative stress markers. The concentration of GSH as an antioxidant parameter was determined in the homogenate samples and measured with spectrophotometric. Data were expressed as μmol/gr tissue.^17^ Malondialdehyde (MDA) as a lipid peroxidation marker was assessed by thiobarbituric acid (TBA) and the colorimetric absorbance was measured at 535 and 520 nm in a spectrophotometer. Results were expressed as nmol/gr tissue.^18^

### 2.6 Histopathological assay

The liver samples were fixed in 10% neutral‐buffered formalin solution, dehydrated in alcohol series, embedded in paraffin, and then sections with 5 mm thickness were stained with H&E (hematoxylin and eosin). The liver slides were evaluated blindly by histologist by light microscopy at 400X magnification (Olympus light microscope, Japan). For semi‐quantitative evaluation of liver damage, histological photomicrographs were examined by the liver scoring system. In this study, the extent of sinusoidal dilatation, inflammatory cell infiltration, congestion, degeneration, the proliferation of Kupffer cells, and cytoplasmic vacuolization were studied. For each mouse, five sections, and in each section, 10 fields were randomly assessed. Data were scored as 0 (normal), 1 (mild), 2 (moderate), or 3 (severe).^19^

### 2.7 Immunohistochemical assay

An immunohistochemical examination was done according to the guidance kit company (Abcam Company). Slides were deparaffinized with xylene and rehydrated in alcohol series, endogenous peroxidase activities were blocked by 0.3% H_2_O_2_ in methanol (30 min). Then, tissue sections were incubated with primary antibodies (anti‐caspase‐3 rabbit polyclonal antibody, 1:100 in PBS, v/v, Abcam, lat: GR224831‐2) at 4°C overnight. After incubation with secondary antibody conjugated with horseradish peroxidase (Mouse and Rabbit Specific HRP/DAB, Abcam, Lat: GR2623314‐4) for 2 h, immunohistochemical staining was carried out by incubation with diaminobenzidine tetrahydrochloride for 5 min. Then, the slides were dehydrated and mounted.^20^ The primary antibody was omitted for negative controls. For the quantitative analysis, immunohistochemical photomicrographs were estimated by densitometry using MacBiophotonics ImageJ 1.41asoftware in 5 fields/each section in all groups. The positive staining severity was assessed as the ratio of the stained area to the entire field assessment.

### 2.8 Statistical analysis

The results were expressed as mean ± SD. The Kolmogorov–Smirnov **(**K**‐**S**)** normality test was used in order to evaluate the normality of the data. All statistical comparisons were performed by one‐way ANOVA followed by Tukey test. Scoring data analyzed by Kruskal–Wallis H Test between groups. *p*‐value less than .05 (*p* < .05) was considered statistically significant (Prism software).

## 3 RESULTS

### 3.1 Effects of GLZ on oxidative stress markers of liver tissue in CP‐treated mice

**FIGURE 2:**
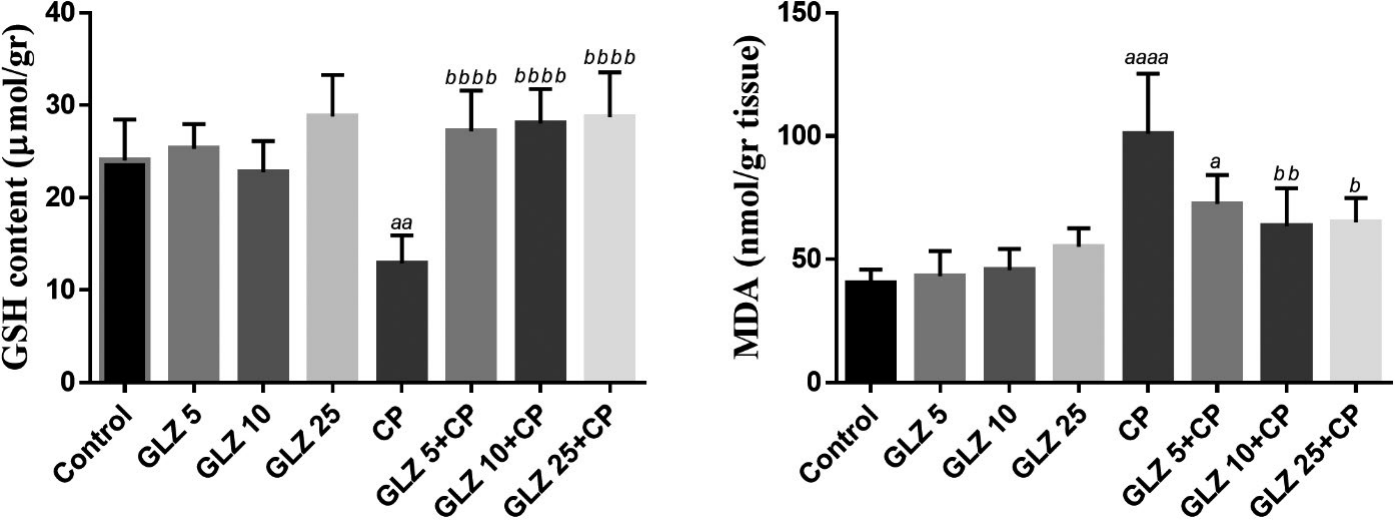
Effects of gliclazide (GLZ) on CP−induced oxidative stress. The data presented are mean ± SD. *a* vs. control group, *b* vs. CP group. GLZ: Gliclazide with 3 doses of 5, 10 and 25 mg/kg; CP: Cisplatin

Figure 2 summarizes the data of the tissue oxidative stress parameters. As shown in Figure 2, there was no difference between the control and the three doses of GLZ groups. The mean level of GSH in the liver tissue of CP‐treated mice was decreased statistically when compared with the control group (*p* < .01). On the other hand, GLZ administration at all three doses increased significantly GSH contents in CP‐injected mice. Contrary to serum biochemical results, tissue biochemical findings showed no significant difference in the three doses of GLZ. The results showed that the hepatoprotective effect of GLZ was not dose‐dependent. There were no statistically significant differences between the different doses of GLZ.

Free radicals and oxidative stress cause lipid peroxidation. The liver MDA level in CP‐treated mice increased significantly compared with the control group. Administration of GLZ in the CP‐treated mice significantly decreased MDA level in liver tissues. GLZ at a dose of 10 mg/kg was more effective in reducing the MDA level (*p* < .01) in CP‐treated mice.

### 3.2 Effect of GLZ on serum liver function enzymes in CP‐treated mice

The effects of CP, GLZ, and their combination of liver function enzymes (AST, ALT, and ALP levels) are shown in Table 1. In this study, CP‐treated mice showed a significant increase in serum AST, ALT, and ALP levels compared with the control group (*p* < .0001). However, administration of GLZ with 5, 10, and 25 mg/kg decreased significantly the serum liver enzymes in the CP‐treated mice. This decrease was dose‐dependent. For ALT and AST markers, a dose of 10 and 25 mg/kg was more effective than the other dose.

**TABLE 1:** Effect of GLZ and CP on serum liver function enzymes in the all groups

Groups	AST U/L	ALT (U/L)	ALP (U/L)
Control	118 ± 11.6	52.8 ± 7.97	151 ± 29.2
GLZ (5 mg/kg)	110 ± 8.91	50.0 ± 6.63	154 ± 21.1
GLZ (10 mg/kg)	117 ± 9.71	48.8 ± 5.44	153 ± 18.1
GLZ (25 mg/kg)	118 ± 7.85	51.3 ± 5.56	146 ± 14.8
CP	183 ± 19.7 *aaaa*	84.3 ± 15.3 *aaaa*	288 ± 48.6 *aaaa*
CP+GLZ (5 mg/kg)	147 ± 14.5 *b*	63.0 ± 2.94 *b*	229 ± 37.9 *a*
CP+GLZ (10 mg/kg)	140 ± 12.0 *bb*	59.5 ± 5.8 *bb*	221 ± 42.8 *b*
CP+GLZ (25 mg/kg)	140 ± 14.9 *bb*	61.8 ± 2.22 *bb*	207 ± 23.6 *b*

All values are expressed as mean ± SD. *a* vs. control group, *b* vs. CP group. GLZ: Gliclazide with 3 doses of 5, 10, and 25 mg/kg, CP: Cisplatin.

### 3.3 Effect of GLZ on histopathology of liver tissue in CP‐treated mice

**FIGURE 3:**
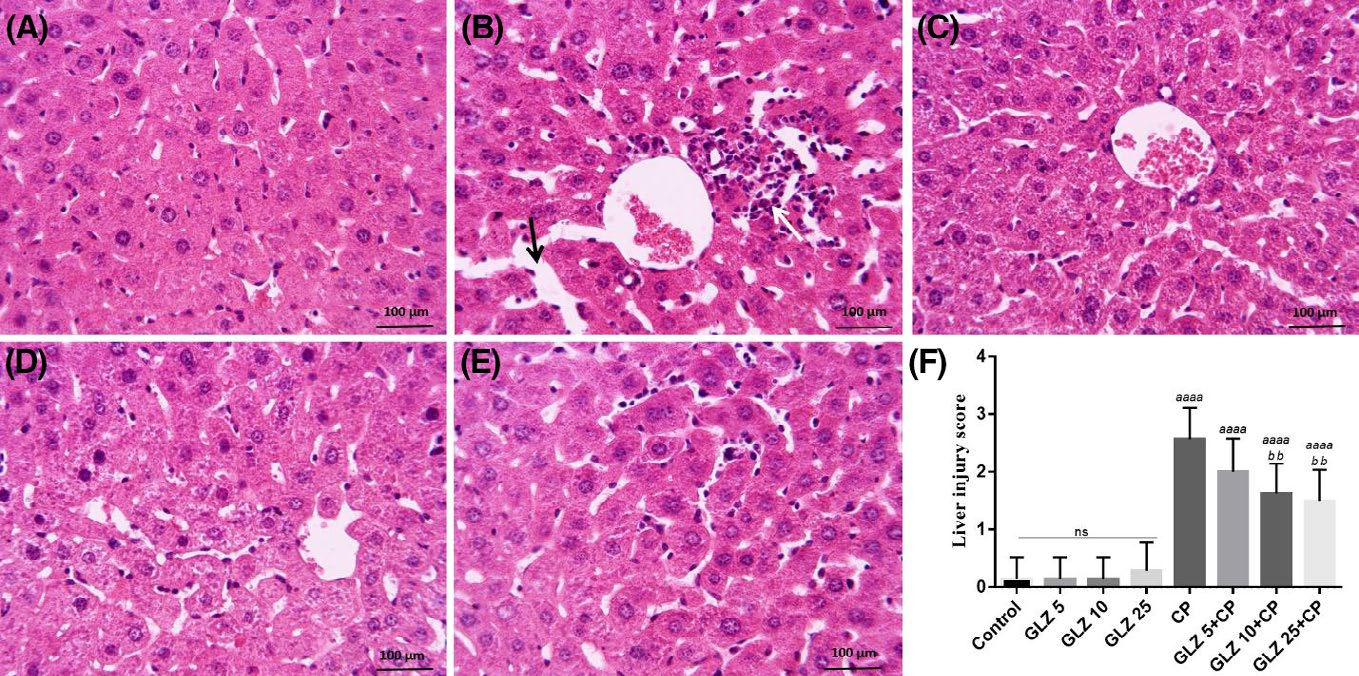
Photomicrographs showed the effect of GLZ treatment and CP on the architecture of the liver in all groups. (A) Control, (B) CP; (C, D), and (E) GLZ (5, 10, and 25 mg/kg, respectively). Normal structure in the control group, infiltration of inflammatory cells (white arrow), dilation sinusoids (black arrow), and hemorrhage of central vein in the CP group are quite obvious. GLZ administration improves these alterations. (F) Injury scores in liver tissue. The data presented are mean ± SD. *a* vs. control group, *b* vs. CP group. GLZ: Gliclazide; CP: Cisplatin. Hematoxylin and Eosin (H & E) staining, Magnification: ×40; Scale bar = 100 μm

Histopathological micrographs in all groups are shown in Figure 3. Liver sections showed that control (Figure 3A) and GLZ (5, 10, and 25 mg/kg) groups had an entirely normal histological appearance. Liver in the CP‐treated mice showed degeneration and eosinophilic cytoplasm in the hepatocytes, dilatation of sinusoids, congestion of central vein, and proliferation of mononuclear white cells and Kupffer cells (Figure 3B). Histopathological finding of liver tissues in GLZ+CP groups showed less damage compared to the CP alone group. Among GLZ+CP groups, GLZ at a dose of 10 and 25 mg/kg showed more effective in the improvement of tissue structure as compared to dose 5 mg/kg (Figure 3C–E).

Furthermore, semi‐quantitative evaluation of liver damage with the scoring system showed that CP administration caused a significant increase in liver injury score (0.29 ± 0.49) compared with control and GLZ alone groups (0.14 ± 0.38). This change with GLZ administration was reduced in CP‐treated mice (Figure 3F).

### 3.4 Effect of GLZ on immunoreactivity of caspase‐3 in the liver tissue in CP‐treated mice

**FIGURE 4:**
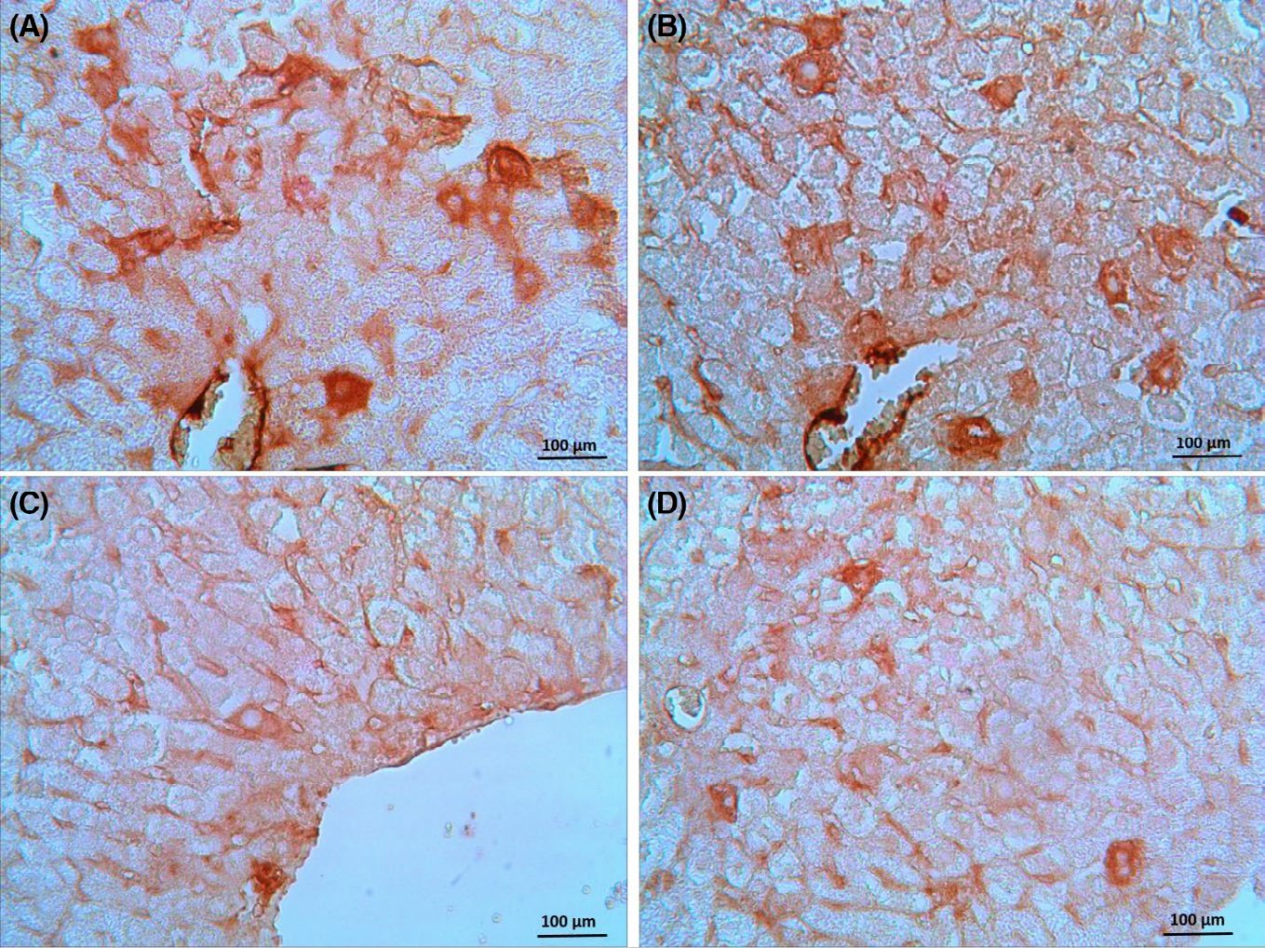
Immunohistochemical staining of caspase−3 in mice liver: (A) CP group with a significant increase in caspase−3 immunoreactivity in the cytoplasm of hepatocytes. (B, C and D) GLZ (With 5, 10, and 25 mg/kg doses, respectively) + CP groups with a significant reduction in caspase−3 immunostaining. Brown color represents caspase−3 positive cells. (Magnification ×40), GLZ; Gliclazide, CP; Cisplatin

Photomicrographs of the immunohistochemical assay are shown in Figure 4. The positive reactions were shown in liver cells with brown color. Caspase‐3 immunoreactivity certified in CP‐treated mice. The more positive reaction was seen in hepatocytes around the lobular central vein. The intensity of the brown color, which indicates the activity of caspase‐3 in hepatocytes, decreased with the administration of GLZ in CP‐treated mice. Of the three doses of GLZ, 10 and 25 mg/kg doses were more effective in reducing apoptosis. The immunoreactivity of caspase‐3 was similar in the control and GLZ alone groups.

**FIGURE 5:**
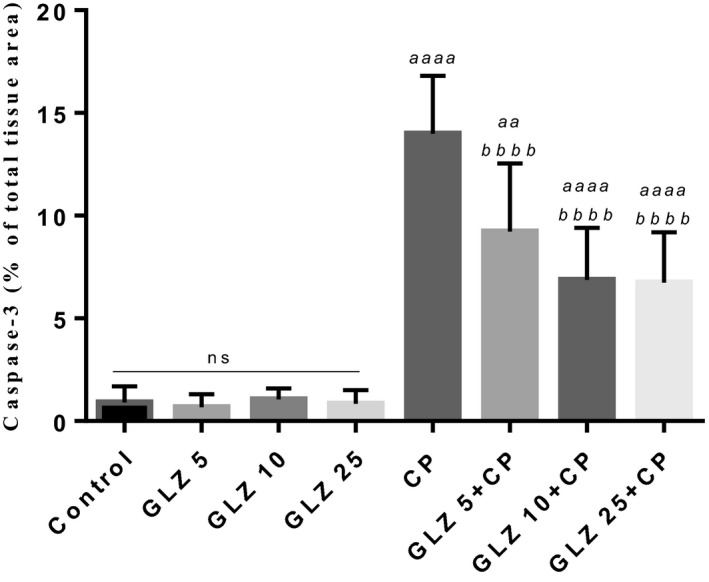
Densitometry analysis of immunohistochemical staining for caspase−3 in the liver tissue. The immunoreactivity level of caspase−3 in the CP alone group increased significantly compared to the control group. Data are presented as Mean ± SD and are a percentage of total tissue area. *a* significant vs. control group, *b* significant vs. CP group. GLZ; Gliclazide, CP; Cisplatin

Figure 5 introduces the immunohistochemical detection of caspase‐3 in the liver tissue. The more immunoreactivity for caspase‐3 was authenticated by semi‐quantitative analysis in the CP‐treated mice (13.99 ± 2.81) compared with the control and GLZ alone groups. Administration of gliclazide in all three doses with cisplatin decreased the severity of the immunoreactivity of caspase‐3, and doses of 10 and 25 mg/kg of GLZ were more effective.

## 4 DISCUSSION

Cisplatin is an anti‐neoplastic agent for solid organ tumors has side effects on most organs including hepatotoxicity.^21^, ^22^ In this study, we showed that CP can significantly induce hepatotoxicity in mice confirmed by elevations in serum liver enzyme activities and histopathological changes. GLZ improved these parameters. The protective effects of GLZ are attributed to a decrease in oxidative stress, suppressing caspase‐3 immunoreactivity.

CP is a small molecule that easily passes through the cell membrane and reaches the cell nucleus and changes the structure of DNA. Although the pathophysiological mechanism of CP‐induced hepatotoxicity is not yet well understood, oxidative stress and apoptosis are important inducers of liver injury.^5^ Researchers have shown that CP increases MDA as a lipid peroxidation marker and reduces GSH as an antioxidant marker in liver tissues.^23^ In oxidative stress, deficiencies occur in the overproduction of reactive oxygen species or the antioxidant system.^24^ GLZ at a low dose has antioxidant activity and can scavenge free radicals.^25^ Our data suggested that GLZ with this potential, protects hepatotoxicity via mitigating oxidative stress and apoptosis in the CP‐treated mice. These findings were consistent with the study of others.^26^ Similar to our findings, Alp et al. showed that GLZ can reduce the amount of MDA in the peripheral and central nervous system of diabetic rats.^9^ GLZ is also able to improve oxidative stress‐induced tissue damage in diabetic patients by reducing 8‐isoprostane as a marker of lipid oxidation and by increasing antioxidant parameters such as TPAC, SOD, and thiols.^27^

In this study, a single dose of CP (10 mg/kg) induced hepatotoxicity. The hepatocellular damage was demonstrated by a significant increase in liver enzymatic markers such as serum ALT, AST, and ALP. Other experimental studies have confirmed these changes associated with hepatotoxicity.^28^ Our serum biochemical results showed that GLZ had a protective effect on liver tissue and reduced the amount of these enzymes by reducing oxidative stress. Patients with type 2 diabetes are at risk for liver damage and subsequently elevated liver enzymes. Belcher has shown that GLZ intake in diabetic patients is able to reduce liver enzymes.^29^ Alsharidah et al. also showed that, the combined metformin/GLZ therapy was able to reduce liver enzymes in diabetic patients.^11^

This study showed that CP led to histopathological changes in liver structure such as sinusoidal dilatation, congestion, central vein dilatation, parenchymal inflammation, hepatocyte cytoplasmic vacuolation, the proliferation of Kupffer, and biliary duct cells in the CP‐treated mice. CP also leads to pathological changes in the liver of cancer patients by inducing oxidative stress.^30^ The protective effect of GLZ with antioxidant property, reducing the MDA and increasing GSH, was investigated on the tissue structure of the peripheral and central nervous system, blood, vascular, and kidney tissue in diabetic experimental models.^9^, ^10^, ^31^, ^32^ GLZ is also metabolized within hepatic microsomes, and two human studies reported that the use of GLZ for a long time leads to hepatitis (acute necro‐inflammation of the liver).^12^, ^13^ In this study, the protective effect of GLZ on liver tissue structure was demonstrated. Our data showed that GLZ at all three selected doses improved CP‐induced liver damage. However, this effect was more effective at doses of 10 and 25 mg/kg as compared to the dose of 5 mg/kg. In the scoring system, the highest score was also for the CP group. And of the three GLZ+CP groups, the dose of 25 mg/kg had the lowest score.

Apoptosis, mitochondrial alterations, and DNA damage are some of the complications of diabetes that are caused by oxidative stress. GLZ by having antioxidant properties could reduce oxidative stress in diabetic patients.^14^, ^33^ Alsharidah has shown the effect of GLZ on some markers of hepatic function.^11^ In this study, we have shown that GLZ can modulate the toxic effect of CP on liver tissue by reducing oxidative stress. Apoptotic signals such as Bax, Bcl‐2, and caspase‐3 are important factors that are expressed in apoptosis.^34^ CP by inducing oxidative stress activates the caspase‐3 apoptotic pathway by inducing oxidative stress.^30^ On the other hand, Wu et al. showed that GLZ decreases the expression of caspase‐3, Bax, and Bcl‐2 levels by reducing oxidative stress in the diabetic model.^35^ Sliwinska et al. showed that GLZ reduced apoptosis in both cancer cells and healthy cells by reducing ROS,^7^ and proved that GLZ had no effect on human normal cells, but it may stimulate DNA repair pathways activation in cancer cells.^36^ Our data suggest GLZ inhibits the activity of caspase‐3 in healthy tissues. In this study, GLZ decreased the immunoreactivity of caspase‐3 in the hepatocyte of CP‐treated mice, and increasing its dose to doses 10 and 25 mg/kg played a more effective role in reducing of immunoreactivity of caspase‐3. This immunohistochemical detection (anti‐inflammatory effect) also authenticated histological results.

Furthermore, we proved the effect of antioxidants in reducing liver toxicity caused by cancer drugs, and confirmed the protective effect of GLZ against DNA damage induced by irradiation in human blood lymphocytes^37^ and in T2DM ^14^ patients. We proved that treatment with GLZ increases the antioxidant defense and reduces hepatotoxicity. These results explain a possible short‐term effect of GLZ against CP‐induced hepatotoxicity. This study has limitations, consisting of the evaluation of other markers of apoptosis (Bax and Bcl‐2) and inflammation (interleukins and NF‐kB). In a clinical study, it also recommended that GLZ be used at the usual dose in cancer patients before starting cisplatin administration.

In conclusion, cisplatin‐induced liver injury is associated with oxidative stress (increased MDA and decreased GSH), disruption of tissue structure, and apoptosis. GLZ treatment improved liver function and histological damage by suppressing oxidative stress and apoptosis activity.

## CONFLICT OF INTEREST

There is no conflict of interest in this study and publication.

## AUTHORS' CONTRIBUTIONS

Taghizadeh and Mirzaei conducted the experiments. Talebpour, Zargari, and Karimpour performed the data analysis. Talebpour and Hosseinimehr participated in research design and wrote or contributed to the writing of the manuscript.

## Data Availability

All data generated in this study are included in this manuscript.
